# Sotatercept administration in a young infant with severe pulmonary arterial hypertension: A case report

**DOI:** 10.3389/fped.2026.1806079

**Published:** 2026-04-29

**Authors:** Arjith V. Rathakrishnan, Jenna Torgeson, Arij Beshish, Monica C. Bogenschutz, Vivek Balasubramaniam, Luke J. Lamers

**Affiliations:** 1 Department of Pediatrics, University of Wisconsin–Madison, Madison, WI, United States; 2 UW Health Kids–Madison, Madison, WI, United States

**Keywords:** pediatric cardiology, pediatric pulmonary arterial hypertension, pediatrics, pulmonary hypertension, sotatercept

## Abstract

Idiopathic pulmonary arterial hypertension (PAH) in infants is a rare, life-threatening condition characterized by elevated pulmonary artery pressure resulting from endothelial dysfunction, vasoconstriction, and vascular remodeling. The treatment options are limited, and the prognosis is poor. In children, 5-year survival following diagnosis remains suboptimal, with estimates of approximately 74% despite advances in therapy. Unfortunately, many patients continue to experience clinical decline despite optimized medical regimens, highlighting the need for novel therapeutic approaches. Sotatercept, an activin receptor type IIB fusion protein, is a new therapeutic agent that modulates the signaling pathway of the transforming growth factor-beta superfamily. It enhances bone morphogenetic protein receptor type 2 signaling, thereby improving pulmonary vascular remodeling and promoting vasodilation. In adult clinical trials, sotatercept has demonstrated improvements in pulmonary vascular resistance, functional capacity, and biomarkers such as brain natriuretic peptide, while reducing the risk of clinical worsening or death, and it was recently approved by the FDA for use in adults to improve exercise capacity and the World Health Organization (WHO) functional class. Despite promising results in adults, data on the use of sotatercept in pediatric patients remain extremely limited and nonexistent for children <1 year of age. Herein, we report the first known use of sotatercept in an infant with severe, treatment-refractory PAH. Treatment was associated with clinical and echocardiographic improvement through 1 year of follow-up, supporting the potential role of sotatercept as an adjunctive therapy in pediatric PAH.

## Introduction

To our knowledge, this is the first case of a patient receiving sotatercept at such a young age.

### Case description

The patient was born at an estimated 36 weeks of gestation, was small for gestational age (1,810 g), and had intrauterine growth restriction and no prenatal care. Maternal history was limited, although there was no reported use of selective serotonin reuptake inhibitors or nonsteroidal anti-inflammatory drugs during pregnancy. Our patient was initially on room air; however, on day of life 9, she decompensated, requiring non-invasive positive pressure respiratory support. An echocardiogram on day of life 10 revealed significantly elevated pressure in the right ventricle (RV) and pulmonary artery (PA) in the setting of a moderate-sized patent ductus arteriosus (PDA), a moderate-sized atrial septal defect (ASD), and a small mid-muscular ventricular septal defect (VSD). Clinically, she struggled with respiratory distress, chronic oxygen dependence, and feeding intolerance, prompting her transfer to our facility at 3 weeks of age for further evaluation and management. She was found to have multiple congenital anomalies, including a cleft palate, a cerebral artery anomaly, severe right pelvocaliectasis, and hemangiomas. Genetic testing, including a microarray and whole-exome sequencing, yielded negative results. A CT scan revealed right heart, main, and branch pulmonary artery dilation without any evidence of interstitial lung disease (ILD), pulmonary veno-occlusive disease (PVOD), or pulmonary capillary hemangiomatosis (PCH).

Cardiac catheterization performed at 5 weeks of age revealed a Qp:Qs ratio of approximately 3:1, a systemic RV pressure of 60/6 mmHg, a mean pulmonary artery pressure (mPAP) of 41 mmHg with a pulmonary artery wedge pressure (PAWP) of 7 mmHg in the setting of a descending aorta pressure of 60/27 mmHg, and an indexed pulmonary vascular resistance (PVRi) of ∼4 WUxm2. Given these findings, a transcatheter PDA closure was performed using a 5–4 Piccolo device. Despite this, RV and PA pressures remained systemic on follow-up echocardiograms, with bidirectional shunting across the small muscular VSD and ASD, mild RV hypertrophy, and moderate RV dilation. She was eventually discharged at 9 weeks of age with ¼ liter per minute (LPM) of oxygen via a nasal cannula (NC).

The patient displayed gradual deterioration following discharge, including decreased oral feeding, failure to thrive, and an increasing oxygen requirement with functional class IIIa–IIIb based on the Pulmonary Vascular Research Institute (PVRI) and the WHO functional class of pulmonary hypertension in children (1). At 4 months of age, a repeat cardiac catheterization revealed a Qp:Qs ratio of 1:1, a suprasystemic RV pressure of 86/9 mmHg, a mPAP of 60 mmHg with a PAWP of 12 mmHg in the setting of a descending aorta pressure of 64/38 mmHg, and a PVRi of ∼10 WUxm2. Inhaled nitric oxide (iNO) testing was performed, and repeat hemodynamics revealed a Qp:Qs ratio of 1.2:1, and a mPAP of 58 mmHg with a PAWP of 12 mmHg, indicating minimal improvement in her PVRi to 9 WUxm2. The distal pulmonary vasculature was markedly abnormal, with tortuous, plump, and pulsatile distal vessels and minimal distal capillary filling. A repeated CT scan was again negative for ILD, PVOD, or PCH. As her intracardiac shunts were not considered to be the primary driver of her pulmonary vascular disease and no other obvious cause could be identified, she was diagnosed with idiopathic precapillary pulmonary arterial hypertension (PAH) (2–4). Postprocedural recovery was difficult and included prolonged intubation and evidence of pulmonary hypertensive crises. Medical therapy with sildenafil and bosentan was initiated, and she ultimately required intravenous treprostinil to wean off iNO. The treprostinil dose was increased to 44 ng/kg/min over 10 days. Subsequent echocardiographic assessments continued to reveal systemic to suprasystemic RV/PA pressures with severe RV dilation, moderate RV hypertrophy, and mildly diminished RV systolic function. She was eventually extubated and weaned to her baseline of 1 LPM of oxygen via NC while tolerating postpyloric tube feeds. She was discharged at 5 months of age on sildenafil 1 mg/kg every 6 h, bosentan 2 mg/kg every 12 h, and treprostinil at 64 ng/kg/min.

Despite further treprostinil uptitration to 90 ng/kg/min, an echocardiographic evaluation at 7 months of age continued to show suprasystemic RV pressure, worsening tricuspid regurgitation (TR) (now moderate to severe), and moderately decreased RV systolic function associated with clinical deterioration (desaturation, increased work of breathing, and failure to thrive) and a similar PVRI/WHO functional class of IIIb. The patient was deemed ineligible for a Potts shunt due to her weight and size, and her family declined a lung transplant evaluation. Given the progressive clinical decline despite receiving aggressive PAH therapy, sotatercept was initiated as an off-label, compassionate-use intervention after informed consent was obtained from the family. She was admitted for observation during the initiation of sotatercept and was discharged after 5 days with no adverse events.

### Therapeutic intervention

Given the absence of published pharmacokinetic or pharmacodynamic data for sotatercept in infants or young children at the time of treatment initiation, the dosing for this patient was derived through extrapolation from available adult clinical trial data and the anticipated pediatric dosing strategy under investigation. Sotatercept is a recombinant fusion protein with linear pharmacokinetics, a long half-life, and clearance mechanisms that are not primarily dependent on hepatic cytochrome P450 metabolism or renal elimination, supporting weight-based dose extrapolation across age groups (5). An initial dose of 0.3 mg/kg administered subcutaneously every 3 weeks, with a planned escalation to a target dose of 0.7 mg/kg, was selected based on the safety, tolerability, and titration parameters established in phase III adult trials, including STELLAR (5–8). At the time of dosing, the MOONBEAM trial (NCT05587712) had not yet enrolled younger pediatric patients; however, the trial's planned weight-based dosing strategy provided a reference point, as it was designed to mirror adult exposure while accounting for pediatric physiology. This approach is consistent with standard pediatric pharmacotherapy principles for biologic agents, wherein dosing is often extrapolated from adult data in the context of rare diseases and limited pediatric experience.

Given the infant's young age, low body weight, and limited subcutaneous tissue volume, additional considerations included injection volume and administration feasibility. Sotatercept was prepared according to the manufacturer’s specifications and reconstituted with sterile water to a final concentration of 50 mg/mL (standard manufacturer’s instructions from 1 vial of Sotatercept kit). Individualized dosing volumes, ranging from 0.04 to 0.13 mL (2–6.3 mg), were calculated based on weight and administered using an insulin syringe with an attached needle to ensure accurate dosing and optimize subcutaneous tolerability. The infant was given 0.3 mg/kg for the initial two doses, which was then increased to 0.7 mg/kg for the third and subsequent doses.

Close safety and efficacy monitoring was incorporated to mitigate the uncertainty related to extrapolated dosing and dose titration. Laboratory monitoring included a complete blood count, a comprehensive metabolic panel, and a brain natriuretic peptide (BNP) obtained prior to each of the first five doses and then every 1–2 months over the course of a year to assess hematologic, hepatic, and renal effects. Clinical assessments and echocardiograms were performed at weeks 3, 9, and 15 following therapy initiation and continued every 1–2 months over the course of a year to evaluate the treatment response, cardiac function, and pulmonary hemodynamics over time.

### Follow-up and outcomes

**Figure 1:**
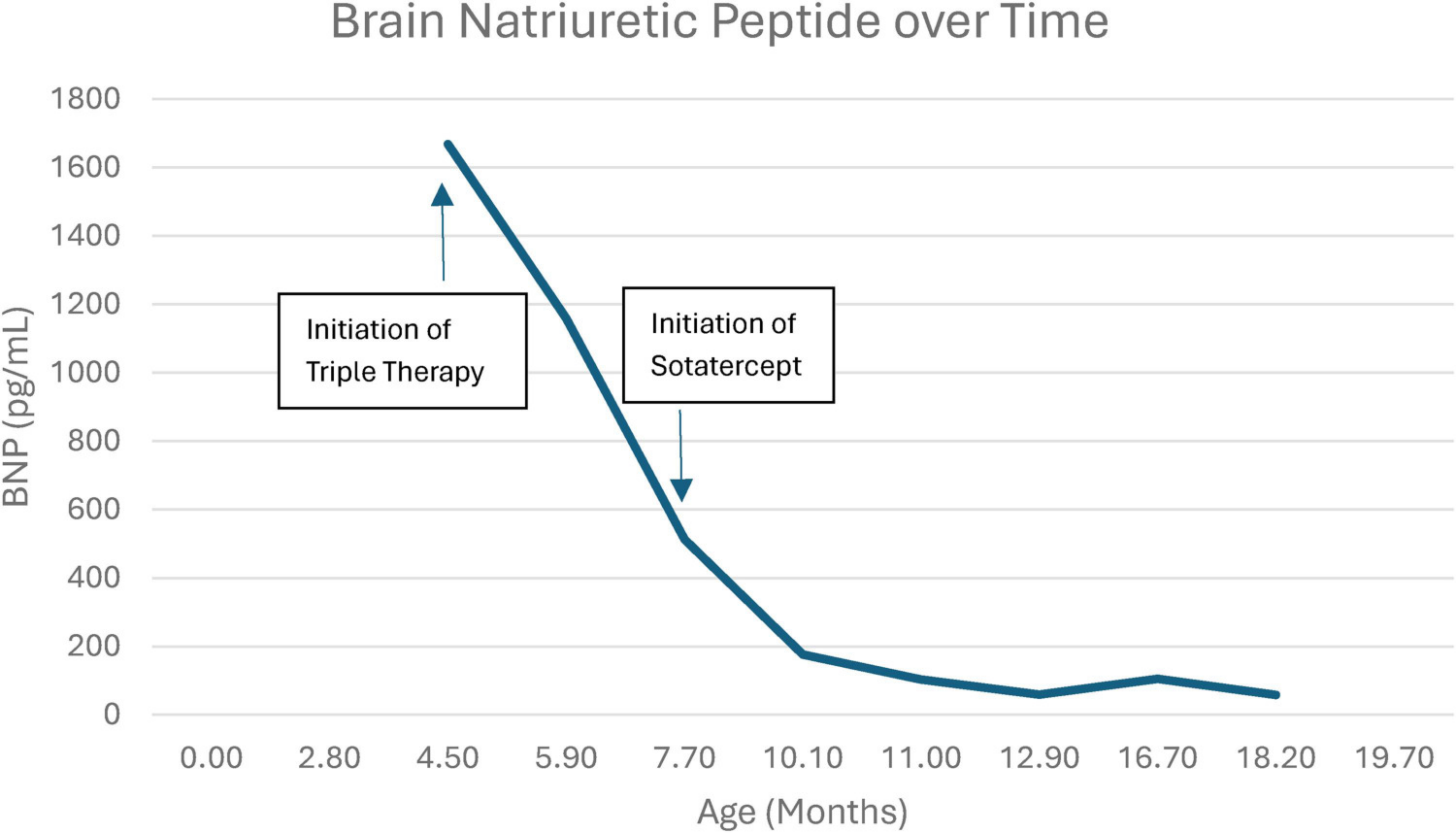
BNP trend over time, showing sustained improvement following the initiation of sotatercept.

Within the first few weeks of initiating sotatercept, the patient demonstrated clinical improvement, with decreased work of breathing, increased alertness, and improved oxygen saturation. Repeat echocardiogram results showed improvement in the TR (from moderate to severe to moderate) and the TR maximal velocity (v-max) from 5.7 to 4.6 m/s. Two months after starting sotatercept, her RV dilation had improved from severe to moderate, the now tiny VSD that demonstrated right-to-left shunting immediately prior to initiating sotatercept was left to right with a v-max of 1.8 m/s, and her tricuspid annular plane systolic excursion (TAPSE) improved from 1.41 to 1.59 cm. Subsequent echocardiograms up to 12 months following the initiation of sotatercept revealed a stable RV size, low-normal RV systolic function, TR v-max between 4 and 4.5 m/s, and left-to-right shunting through the VSD with a v-max of 2.3 m/s. She displayed an improved functional class (PVRI/WHO class II) and clinical stability with no worsening during outpatient visits. The patient’s weight at the initiation of sotatercept was 5.98 kg, representing the <1st percentile for age. She demonstrated consistent favorable somatic growth following the initiation of sotatercept, with her most recent weights ranging between the 10^th^ and the 25th percentiles for age. BNP levels improved from 513 pg/mL prior to starting sotatercept to as low as 40 pg/mL within ∼2 months following initiation, with the most recent value at 58 pg/mL (Figure 1). Her hemoglobin levels increased from 9.7 g/dL immediately prior to the initiation of sotatercept to a peak level of 15.4 g/dL following 6 months of therapy and then stabilized between 12 and 13 g/dL at 9- and 12-month follow-ups. Her baseline hemoglobin was ∼11 g/dL in the months prior to starting sotatercept, and as her levels increased gradually, no doses of sotatercept were administered. Her platelet count remained stable while receiving sotatercept. No significant adverse events or abnormal laboratory findings were observed during the first year of therapy. Table 1 highlights the clinical, echocardiographic, and laboratory data observed during her course of treatment. She remained on a baseline of 1 LPM of oxygen via NC, with oxygen saturation >94% (80s without oxygen). Over the 12 months following sotatercept initiation, she was transitioned to ambrisentan 2.5 mg (0.27 mg/kg) once daily and tadalafil 1 mg/kg once daily while remaining on treprostinil at 116 ng/kg/min. Sotatercept dosing was adjusted for weight gain and is currently at 6 mg (0.13 mL), which is 0.7 mg/kg every 3 weeks.

**Table 1:** Longitudinal clinical, echocardiographic, and laboratory data during treatment of severe pediatric pulmonary hypertension.

Age/timepoint	BNP (pg/mL)	TR velocity (m/s)	Hemoglobin (g/dL)	Platelets (K/µL)	Therapies/events	WHO/PVRI class
4 months	1,668	—	10.8	303	Repeat catheterization	IIIa–IIIb
5 months	1,458	5.0	11.7	252	Treprostinil initiated	IIIa–IIIb
6 months	453	5.4	12.3	331	Treprostinil uptitration	IIIb
7 months	513	5.7	9.7	252	Sotatercept initiated	IIIb
9 months	40	4.6	15.4 (peak)	313	___	IIIa–IIIb
12 months	—	4.3	14.8	414	—	II
18 months	58	4.3	12	240	Functional improvement	II

Values from diagnosis through prostacyclin initiation and subsequent sotatercept therapy, demonstrating improvements in BNP, TR velocity, and functional class.

## Discussion

In this infant with severe PAH, the addition of sotatercept to an optimized, triple-drug regimen resulted in sustained improvements in both functional class and echocardiographic parameters, with no significant adverse events at the 1-year follow-up. The observed response suggests the potential benefit of pathway-directed therapy in select pediatric patients with refractory disease. Sotatercept is a first-in-class fusion protein that restores the balance of the transforming growth factor-beta/bone morphogenetic protein receptor type 2 pathway, a key regulator of pulmonary vascular remodeling (5–7). By inhibiting abnormal vascular remodeling, inflammation, and smooth muscle proliferation, sotatercept targets mechanisms not directly addressed by conventional PAH therapies. This mechanistic difference provides a rationale for its use as an adjunctive therapy in patients with progressive disease despite maximal standard treatment.

This case illustrates that sotatercept may provide additional benefits to young pediatric patients with progressive PAH who have a suboptimal response to existing regimens, and it highlights the challenges of medication administration to very small children. In infants, factors such as limited subcutaneous tissue, dosing precision, small allowable injection volumes, and caregiver comfort with administration techniques must be considered for evaluating safety and feasibility. The low dose required the use of a concentrated product, necessitating careful preparation and handling to ensure accurate delivery and reduce the risk of dosing errors.

While these findings are encouraging, the extrapolation of adult dosing to infants remains empirical, and the long-term safety profile in pediatrics is unknown. The ongoing MOONBEAM trial will be critical to defining appropriate dosing, safety, and efficacy in neonates and young children; however, such trials often exclude younger and most critically ill patients, underscoring the importance of early experiences to inform interim clinical decisions. This report highlights the potential role of sotatercept as an adjunct in the treatment of severe, refractory pediatric PAH and documents medication administration strategies in very small children.

## Data Availability

The original contributions presented in the study are included in the article/Supplementary Material, and further inquiries can be directed to the corresponding author.
